# Non-communicable Disease-Related Sustainable Development Goals for 66 Belt and Road Initiative Countries

**DOI:** 10.34172/ijhpm.2022.6172

**Published:** 2022-10-29

**Authors:** Lin Chen, Donghui Duan, Liyuan Han, Lu Xu, Sixuan Li, Yuwei Zhang, Wei Feng, Qinghai Gong, Angela E. Micah, Ruijie Zhang, Shiwei Liu, Hui Li

**Affiliations:** ^1^Ningbo No. 2 Hospital, Ningbo, China.; ^2^Ningbo Municipal Center for Disease Control and Prevention, Ningbo, China.; ^3^Department of Global Health, Ningbo Institute of Life and Health Industry, University of Chinese Academy of Sciences, Ningbo, China.; ^4^Panjin Municipal Center for Disease Control and Prevention, Panjin City, China.; ^5^Fenghua District Center for Disease Control and Prevention, Ningbo, China.; ^6^Department of Health Metrics Sciences/Institute for Health Metrics Chen et al International Journal of Health Policy and Management, 2022, x(x), 1–13 13 and Evaluation, University of Washington, Seattle, WA, USA.; ^7^Division of Chronic Disease and Aging Health management, Chinese Center for Disease Control and Prevention, Beijing, China.

**Keywords:** Non-communicable Disease, Sustainable Development Goals, Belt and Road Initiative, Burden of Disease Study

## Abstract

**Background:** Since 2015, the Global Burden of Disease Study (GBD) has measured progress in achieving health-related Sustainable Development Goals (SDGs) annually worldwide. Little is known about the status and attainment of indicators of non-communicable diseases (NCDs) by 65 countries from the Belt and Road Initiative (BRI) proposed by China in 2013.

**Methods:** Data from GBDs were used to estimate 24 NCD-related SDG indicators in BRI countries from 1990 to 2017. Each indicator was scored from 0 to 100 to compare multiple indicators over the study period. The natural log of the annual change in each location and year and weighted annual rates of change were used to generate projections for 2030. National-level estimates were determined by socio-demographic index (SDI) quintiles in BRI countries with more than 1 million inhabitants.

**Results:** In 2017, the median overall score of NCD-related SDG index for the 66 BRI countries was 60 points, ranging from 29 points in Afghanistan to 84 points in Israel. More than 80% of countries achieved the SDG 2030 maternal mortality (MM) rate target in 2017, and the national skilled birth attendance rate was above 99% in more than 59% countries. However, none of the BRI countries achieved the goal for children’s overweight, modern methods of contraception, and universal health coverage. It was predicted that 80.4% of NCD-related SDG targets would be achieved in these countries by 2030. The overall score of NCD-related SDG index were positively associated with SDI quintiles.

**Conclusion:** For many indicators, the achieved progress in many countries is less than the annual rate necessary to meet SDG targets, indicating that substantial efforts need to be made in the coming years. Progress should be accelerated through collaborations between countries, implementation of NCD prevention and control strategies, and monitoring of inequalities in NCD-related SDGs within populations.

## Background

**Key Messages**

** Implications for policy makers**

- The health status of the population varies greatly by countries. Therefore, establishing long-term cooperation mechanisms and health information platforms, and strengthening health policy communication are fundamental.
- Improving counseling and coordination, and advice to policy-makers in bilateral health cooperation and multilateral health governance are vital.
- Encouraging academic institutions and experts to share experiences on health policy research and activities.
- Strengthening the monitoring of the health condition of the population and establishing regional disease surveillance and early warning systems.

** Implications for the public**

Since 2015, the Global Burden of Disease Study (GBD) has measured progress in achieving health-related Sustainable Development Goals (SDGs) annually worldwide. However, little is known about the status and attainment of indicators of non-communicable diseases (NCDs) by 66 countries from the Belt and Road Initiative (BRI) proposed by China in 2013. In this study, data from GBDs were used to estimate 24 NCD-related SDG indicators in BRI countries from1990 to 2017. We found that the median overall score of NCD-related SDG index for the 66 BRI countries was 60 points, ranging from 29 points in Afghanistan to 84 points in Israel, indicating a large variability. For many indicators, the achieved progress in many countries is less than the annual rate necessary to meet SGD targets, indicating that substantial efforts need to be made in the coming years. Progress should be accelerated through collaborations between countries, implementation of NCD prevention and control strategies, and monitoring of inequalities in NCD-related SDGs within populations.

In 2013, China proposed the Belt and Road Initiative (BRI) to promote trade, infrastructure development, and commercial partnerships among 66 countries in Asia, Africa, and Europe.^1,2^ The initiative aims to build a community of shared interests, goals, and responsibilities based on mutual political trust, economic integration, and cultural inclusiveness.^3^ The global health perspective was formally included in BRI in 2015 and was firmly established during the first BRI Forum for International Cooperation in Beijing in May 2017, where plans for a Health Silk Road were announced and endorsed by beneficiary countries.^3-6^

 In the past few years, China has become more proactive in global health governance. Meanwhile, the World Health Organization (WHO) has welcome China’s efforts to integrate health into its ambitious development agenda and has been working with the country to explore opportunities and synergies for collaboration.^7^ Early efforts have included participating insetting the WHO agenda (on essential and traditional medicine and universal health coverage) and prioritizing chronic disease treatment, drug innovation, and social determinants of health in other countries.^8^ For instance, the promotion of health security was prioritized by China’s response to the Ebola outbreak and long-term funding.^5^ The second effort was the BRI, which will be accompanied by new multilateral institutional arrangements.^5^ The third initiative as the significant increase in China’s financing of global health through development assistance for health, overseas development assistance, and new investment vehicles.^5^

 In September 2015, the United Nations (UN) General Assembly adopted “Transforming our World: The 2030 Agenda for Sustainable Development,” a resolution outlining a new framework to form the cornerstone of the sustainable development agenda until 2030.^9^ This new framework replaced the Millennium Development Goal (MDG) framework, which expired in 2015, and established Sustainable Development Goals (SDGs), including 17 universal goals and 169 targets.^10^ The SDG initiative is currently in its fourth year, and progress towards the global aspirations of this initiative is gradual and ongoing.^11^ An example includes the WHO’s 13th General Programme of Work (GPW13) for 2019-2023,^12^ which involves an ambitious agenda of measurable goals and interconnected strategies to ensure healthy living and well-being for people of all ages.^13^

 Non-communicable diseases (NCDs) pose an increasing economic and health burden on low- and middle-income countries.^14^ In this respect, the WHO estimated that 42% of premature death aged 30-70 years globally were attributed to NCDs and that 48% of these fatalities occurred in these countries.^11,15,16^ The total number of deaths from NCDs increased by 22.7% (21.5%-23.9%) from 2007 to 2017, representing an estimated 7.6 million (7.2-8.0) additional deaths in 2017 than in 2007.^9^ Although NCD prevention has been apriority by the UN, and evidence-based policies and programs have been developed to target NCDs, a substantial gap remains in implementation.^10^ Therefore, preventing NCDs was proposed as a vital task in GPW13.^17^ The *Lancet* announced that 2018 was the year for action against NCDs,^17^ and the WHO Independent High-Level Commission on NCDs declared in the Time to Deliver^12,18^ that there was no excuse not to act.^11^ These events have paved the way for the actions proposed in the present study.

 The health level of populations across BRI countries varies significantly because of variations in the geographical distribution of populations and health status across continents. With the progress of globalization and the continuous promotion of the BRI, health services and the health status of populations are gradually improving in BRI countries.^19-21^ However, the prevention of NCDs in BRI countries is facing several challenges, such as inadequate healthcare investment,^22^ poor health literacy and lack of awareness,^23^ which have left no country untouched and bridges the divide between rich and poor countries.^24^ In GPW13, there were 41 health-related SDGs, of which 25 were associated with NCDs.^13^ The Global Burden of Disease Study (GBD) measured global progress in achieving health-related SDGs since 2015,^10^ which is useful to assess health-related SDG indicators across BRI countries over time in a comparable manner.^11,25^

 Understanding of how past rates of progress translate into future trajectories for the NCD-related SDGs is an important input for decision makers, particularly during these initial years of SDG policy development and implementation. In order to help to address public health challenges of NCDs in BRI countries, we use GBD data to estimate scores for 24 NCD-related SDGs, analyze new challenges in NCD prevention, and propose suggestions to improve health literacy and reduce risk factors.

## Methods

###  Overview of 65 Belt and Road Initiative Countries

 As of 2017, 65 countries (except China) spanning three continents were included in the BRI, including two countries in East Asia, 11 countries in Southeast Asia, seven countries in South Asia, five countries in Central Asia, nineteen countries in West Asia, one country in North Africa, and twenty countries in Central and Eastern Europe (Supplementary file 1, Table S1). The gross domestic product (GDP) of these countries is approximately US$ 23.32 trillion, accounting for 30% of the global GDP.^26^ However, it is of note that 2 low-income countries, 23 lower-middle income countries, 22 upper-middle income countries, and 18 high-income countries were included in the BRI.^27^ Moreover, the number of people living in low-, middle-, and high-income countries is currently 290 million, 2.39 billion, and 300 million, respectively, accounting for 6.7%, 55.2%,and 6.9% of the global population, respectively.^28^

###  Overview of the Global Burden of Disease 2017

 The GBD produces age-, sex-, and location-specific estimates of all-cause and cause-specific mortality, non-fatal outcomes, overall disease burden, risk factor exposure, and attributable burden from 1990 to the current study year using standardized and replicable methods that conform to the Guidelines for Accurate and Transparent Health Estimates Reporting.^10,29^ The GBD 2017 measured progress in achieving 41 health-related SDGs in 195 countries and territories from 1990 to 2017 around the world, including the 66 countries in BRI, projected indicators for the year 2030, and measured global performance.^11^ Therefore, we included data for all 66 BRI countries from the GBD 2017 in this study.

###  Socio-demographic Index 

 The socio-demographic index (SDI) is a composite measure of overall development based on rescaled values of fertility, education, and income, and is used to compare progress in achieving health-related SDGs across quintiles of overall development.^30^ The SDI was updated to include fertility rates among women younger than 25 years rather than total fertility rates in the GBD 2017.^11^ In this study, quintile breaks were generated from the SDI distribution at the national level in BRI countries with more than 1 million inhabitants.^11^

###  NCD-Related Sustainable Development Goal Index

 Health-related SDG data were extracted from the GBD 2015,^10^ and 41 indicators were updated included in the GBD 2017 (population census coverage was excluded because of its binary status and absence of forecasts).^11^ These indicators were created by a preference-weighted approach, in which SDGs represented the expressed preferences of UN member states and therefore assumed that each SDG target should be weighted equally.^11^ The data sources and estimation approaches used in this analysis were detailed in previous publications.^10,11,25^

 In this study, NCD-related SDG indicators were defined as indicators for health outcomes, and environmental, occupational, behavioral, maternal and infant conditions, and metabolic risks with well-established causal connections to NCDs, except for acute infection related, and injuries related factors.^31^ We provided updated estimates for 24 NCD-related SDGs in 66 BRI countries (the score of indicator 17.19.2a census was not included because of its binary status and absence of predictions), and all analyses were based on GBD 2017 methods.^11^ Each indicator was scored from 0 to 100, with 0 being the lowest (worst) observed value and 100 being the highest (best) value, to allow comparisons between indicators from 1990 to 2030.^11^ Conversely, negative indicators, for which lower values were more desirable than higher values, were assigned a value from 0 to 100, reflecting the worst to the best performance.^11^ The applied formula was:


$$Scaled\;score=\frac{\left(observed\;value-worst\;observed\;value\right)\;\times 100}{best\;observed\;value-worst\;observed\;value}$$

where the observed value is the actual value of the country in the current year, and the worst and best observed value is the lowest and highest value of each indicator in BRI countries in the period 1990-2017, respectively.

 We calculated the geometric mean of scaled NCD-related SDG indicators by target, and measured the geometric mean across all NCD-related SDGs to obtain a median total score. The geometric mean allows indicators with very high values to partly compensate for low values on other indicators (referred to as partial substitutability).^10,11^ Furthermore, the minimum value was restricted to 1 when calculating the overall score to reduce the effect of values close to 0.

 Additionally, four newly estimated scores including healthy lifestyle (Lif), healthy pregnancy and infant (P-I), growth and development (G&D), as well as healthy environment (Env) were generated in this study. The Lif index is an overall estimate measure of four scaled NCD-related indicator, which is calculated the geometric mean of overweight, alcohol, smoking and meet the need for family planning (FP) and contraception. The P-I index is an overall estimate measure for four scaled NCD-related indicators, which is calculated the geometric mean of maternal mortality (MM) ratio, skilled birth attendance, under-5 mortality, and neonatal mortality. The G&D index is an estimate measure for two scaled NCD-related indicators, which is calculated the geometric mean of stunning and wasting. The last is Env, an overall estimate measure for six NCD-related indicators, which is calculated the geometric mean of air pollution mortality, water, sanitation, hygiene, household air pollution, and mean PM_2.5_.

###  Projection of NCD-Related Sustainable Development Goals for 2030

 To generate projections for 2030, we used a forecasting method^32^ by calculating the natural log of the annual change in each location and year from 1990 to 2017 and weighted annual rates of change. This modeling framework was used to analyze the relationships between risk factors and other independent drivers of NCDs and better assess the causes of health problems reported in randomized controlled trials and cohort studies.^11^ The targets of some NCD-related SDGs are clearly defined in the UN decision-making document,^33^ whereas other goals were not defined. Moreover, the thresholds of 16 indicators with defined targets were used in this study. We replaced “all popular” or “full coverage” in the target specification with “>99%,” the “elimination” target in the health environment with “<1%,” and “eliminate” goals in epidemics with “<0.5%.” In addition, indicators for undefined targets were not predicted^11^. The target status of each indicator is shown in Table 1.

**Table 1:** Non-Communicable Disease-Related Sustainable Development Goals, Sub-goals, Indicators, and Targets for 2030

	**NCD-Related Indicators**	**Definition**	**2030 SDG Target**
**Goal 2: End Hunger, Achieve Food Security and Adequate Nutrition, and Promote Sustainable Agriculture**			
Target 2.2: By 2030, end all forms of malnutrition; by 2025, achieve internationally-agreed targets on stunting and wasting in children under 5 years of age, and address the nutritional needs of adolescent girls, pregnant and lactating women, and older persons	Stunting (2.2.1)	Prevalence of stunting in children under 5 years of age, %	Eliminate
Target 2.2 (as above)	Wasting (2.2.2a)	Prevalence of wasting in children under 5 years of age, %	Eliminate
Target 2.2 (as above)	Overweight (2.2.2b)	Prevalence of overweight in children aged 2–4 years, %	Eliminate
**Goal 3: Ensure a Healthy Lifestyle and Promote the Well-being for All Ages**			
Target 3.1: By 2030, reduce the global MM rate to less than 70 deaths per 100 000 live births	MM ratio (3.1.1)	Maternal deaths per 100 000 live births	<70/100 000
Target 3.1 (as above)	Skilled birth attendance (3.1.2)	Proportion of births attended by skilled health personnel (physicians, nurses, midwives, or country-specific medical staff [eg, clinical officers]), %	100%
Target 3.2: By 2030, end preventable deaths of newborns and children under 5 years of age, with all countries aiming to reduce neonatal mortality to at least as low as 12 per 1000 live births and under-5 mortality to at least as low as 25 per 1000 live births	Under-5 mortality (3.2.1)	Probability of dying before age 5 years per 1000 live births	Decreased ≥25‰
Target 3.2 (as above)	Neonatal mortality (3.2.2)	Probability of dying during the first 28 days of life per 1000 live births	Decreased ≥12‰
Target 3.4: Reduce premature mortality from NCDs by one-third through prevention and treatment, and promote mental health and wellbeing by 2030	NCDs (3.4.1)	Age-standardized death rate due to cardiovascular disease, cancer, diabetes, and chronic respiratory disease in populations aged 30–70 years per 100 000 population	Reduce by 1/3
Target 3.4 (as above)	Suicide (3.4.2)	Age-standardized death rate due to self-harm per 100 000 population	Reduce by 1/3
Target 3.5: Strengthen the prevention and treatment of substance abuse, including narcotic drugs and alcohol	Alcohol (3.5.2)	Risk-weighted prevalence of alcohol abuse measured by the SEV for alcohol abuse, %	Undefined
Target 3.7: By 2030, ensure universal access to sexual and reproductive healthcare services for FP and education, and the integration of reproductive health into national strategies and programmes	Meet the need for FP and contraception (3.7.1)	Proportion of women of reproductive age (15–49 years) whose need for FP is satisfied with modern methods, % women aged 15–49 years	100%
Target 3.7 (as above)	Adolescent birth rate (3.7.2)	Birth rates among women aged 10–14 years and 15–19 years, number of live births per 1000 women aged 10–14 years and 15–19 years	Undefined
Target 3.8: Achieve universal health coverage, including financial risk protection, access to essential high-quality healthcare services, and access to safe, effective, high-quality, and affordable essential medicines and vaccines for all people	Universal health coverage tracer (3.8.1)	Coverage of universal health, coverage tracer interventions for prevention and treatment services, %	Fully accessible (100%)
Target 3.9: By 2030, substantially reduce the number of deaths and illnesses from hazardous chemicals, and reduce air, water, and soil pollution and contamination	Air pollution mortality (3.9.1)	Age-standardized death rate attributable to air pollution, per 100 000 population	Undefined
Target 3.9 (as above)	WASH mortality (3.9.2)	Age-standardized death rate attributable to unsafe WASH per 100 000 population	Undefined
Target 3.a: Strengthen the implementation of the World Health Organization Framework Convention on Tobacco Control in all countries, as appropriate	Smoking (3.a.1)	Age-standardized prevalence of daily smoking in populations aged 10 years and older, % population aged 10 years and older	Undefined
Target 3.c: Substantially increase health financing and the recruitment, development, training, and retention of the health workforce in developing countries, especially in low- and middle-income countries and small island developing states	Health worker density (3.c.1)	Health worker density per 1000 population, by cadre and summed across cadres	Undefined
**Goal 6: Ensure the Availability and Sustainable Management of Water and Sanitation for All**			
Target 6.1: By 2030, provide universal and equitable access to safe and affordable drinking water for all people	Water (6.1.1)	Risk-weighted prevalence of populations using unsafe or unimproved water sources, as measured by the SEV for unsafe water, %	Eliminate
Target 6.2: By 2030, provide equitable access to adequate sanitation and hygiene for all people and end open defecation, paying special attention to the needs of women and girls and individuals in vulnerable situations	Sanitation (6.2.1a)	Risk-weighted prevalence of populations using unsafe or unimproved sanitation, as measured by the SEV for unsafe sanitation, %	Eliminate
Target 6.2 (as above)	Hygiene (6.2.1b)	Risk-weighted prevalence of populations with poor hygiene (no hand washing with soap), as measured by the SEV for unsafe hygiene, %	Eliminate
**Goal 7: Ensure Access to Affordable, Reliable, and Sustainable Energy for All People**			
Target 7.1: By 2030, ensure universal access to affordable, reliable, and modern energy services	Household air pollution (7.1.2)	Risk-weighted prevalence of ambient air pollution, as measured by the SEV for ambient air pollution, %	Eliminate
**Goal 8: Promote Lasting, Inclusive, and Sustainable Economic Growth, Promote Full Productive Employment and Decent Work for All People**			
Target 8.8: Protect labor rights and promote safe and secure working environments for all workers, including migrant workers, in particular female migrants, and workers in precarious employment	Occupational risk burden (8.8.1)	Age-standardized all-cause disability-adjusted life year (DALY) rate attributed to occupational risk (per 100 000)	Undefined
**Goal 11: Make Cities and Human Settlements Inclusive, Safe, Resilient, and Sustainable**			
Target 11.6: By 2030, reduce the adverse per capita environmental impact of cities, paying special attention to air quality and waste management	Mean PM_2.5_ (11.6.2)	Population weighted mean level of PM_2.5_ (μg/m^3^)	Undefined
**Goal 17: Establish and improve Global Partnerships for Sustainable Development**			
Target 17.19: By 2030, improve existing initiatives to develop measurements of progress on sustainable development that complement gross domestic product, and support statistical capacity building in developing countries	Population census (17.19.2a)	Population and housing census status over the past 10 years	Census in the past 10 years
Target 17.19 (as above)	Death certificates (17.19.2c)	Number of deaths certificates in a vital registration system relative to a country’s total deaths (%)	80% of total deaths

Abbreviations: NCD, Non-communicable disease; SDG, Sustainable Development Goal; MM, Maternal mortality; WASH, Water, sanitation, and hygiene; SEV, summary exposure value; FP, family planning.

 Based on the results of the GBD 2017^11^ and the weight of populations in BRI countries, we estimated the national average of each indicator for 2017. Simultaneously, based on the 2030 target, we determined the annual rate of change required for BRI countries to achieve their goals from 2017 to 2030, and compared with the average annual rate of change from 1990 to 2017 to predict progress in achieving each goal. However, we used average scores of indicators to reduce the heterogeneity in drivers of growth across BRI countries. In addition, forecasts were based on past rates of change; therefore, changes in several factors between the present date and 2030, including health financing, global health priorities, and climate change, could not be determined accurately. Census coverage was not estimated because of its binary nature and the lack of documentation about planned censuses across all countries. In addition, indicators were not estimated by sex or region.

## Results

###  NCD-Related SDGs in BRI Countries From 1990 to 2017

**Figure 1:**
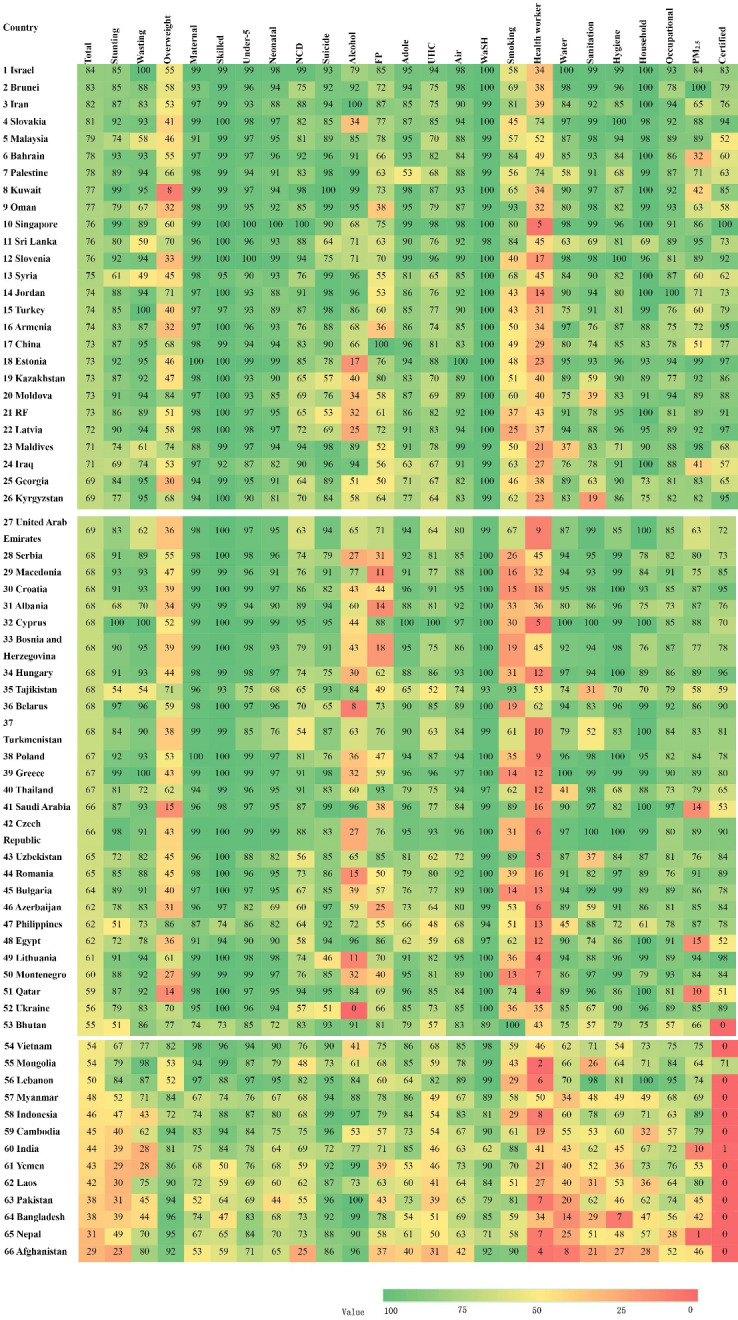
Scores of 24 Non-communicable disease-Related Sustainable Development Goal Indicators in 66 Countries From Belt and Road Initiative Countries in 2017. Abbreviations: NCD, Non-communicable disease; FP, family planning; WASH, Water, sanitation, and hygiene; UHC, universal health coverage.

 The scores of 24 NCD-related SDGs in BRI countries are shown in Figure 1. In 2017, the median overall score of NCD-related SDG index for all 66 BRI countries was 60 points, ranging from 29 points in Afghanistan to 84 points in Israel. China scored 73 points and was ranked No. 17. There was a difference of more than 50 points between Afghanistan and Israel. However, more than 80% of countries achieved the 2030 target for MM rate (<70 deaths per 100 000 live births), and more than 59% achieved skilled birth attendance rate >99% (the target was 100%). Furthermore, of 24 NCD-related SDGs, none of the evaluated countries achieved the goals for children’s overweight (2.2.2b), modern methods of contraception (3.7.1), and universal health coverage (3.8.1).

 Israel had the highest sc ore (84) among all countries, and the scores for nine NCD-related SDGs were>99, including stunting (99.3), malnutrition (99.6), MM (99.5), safe delivery (99.2), under-five mortality rate (U5MR) (99.1), water, sanitation, and hygiene (WASH) mortality (99.9), drinking water (99.8), individual health (99.3), and household air pollution (99.6) (Figure 1).

 With regard to adverse health outcomes and risks, the goal was eliminating stunting, malnutrition, and overweight (Table 2). In this study, the highest scores for stunting were 99.9 in Cyprus, and 99 in Greece and Israel, and the lowest score was 22.8 in Afghanistan. The average total score was 99.3. For malnutrition, the three highest scores were 99.9 in Cyprus, 99.7 in Turkey, and 99.6 in Greece and Israel. The lowest score was 27.9 in India. For overweight, the top three scores were 96.3 in Bangladesh, 94.5 in Nepal, and 94.2 in Cambodia. The lowest score was 8.2 points in Kuwait (Figure 1).

**Table 2:** Predicting the Achievement of NCD-Related SDG Indicators in 2030 on the Basis of Annual Change From 1990 to 2017 Across Countries

**Countries**	**Total Score of SDG Indicators in 2017**	**Annual Rates of Change From 1990 to 2017** ^a^	**Predicted Total Score of SDG Indicators in 2030**	**Target Year for Achieving the Indicator**
Total 66 countries from the BRI	68.0	1.33	80.4	2046
Afghanistan	46.0	2.16	60.7	2053
Albania	80.3	0.91	90.3	2041
Arab	69.0	0.57	74.3	2081
Armenia	75.9	0.80	84.1	2051
Azerbaijan	68.7	1.11	79.3	2050
Bahrain	81.5	1.04	93.2	2036
Bangladesh	59.8	2.18	79.1	2041
Belarus	83.6	0.72	91.7	2041
Bhutan	64.1	2.11	84.0	2038
Bosnia and Herzegovina	76.6	0.64	83.2	2058
Brunei	76.5	0.47	81.4	2072
Bulgaria	77.7	0.37	81.5	2084
Burma	58.4	1.46	70.5	2053
Cambodia	61.9	2.27	82.9	2038
China	80.4	1.53	98.0	2031
Croatia	87.2	0.51	93.2	2042
Cyprus	93.6	0.71	102.6^b^	2026
Czech Republic	88.6	0.67	96.6	2034
Egypt	65.2	1.21	76.2	2052
Estonia	85.5	0.75	94.3	2037
Georgia	71.2	0.36	74.6	2109
Greece	90.9	0.40	95.7	2040
Hungary	84.2	0.66	91.7	2042
India	56.8	1.55	69.4	2053
Indonesia	61.8	1.23	72.4	2056
Iran	76.7	0.85	85.6	2048
Iraq	71.1	0.94	80.2	2053
Islam	89.6	0.52	95.9	2037
Jordan	77.3	1.01	88.1	2042
Kazakhstan	73.1	0.60	79.0	2068
Kuwait	84.8	0.75	93.5	2038
Kyrgyzstan	68.6	0.79	76.0	2064
Laos	53.0	2.75	75.4	2040
Latvia	81.9	0.54	87.8	2053
Lebanon	81.2	1.04	92.9	2036
Lithuania	81.2	0.43	85.9	2064
Macedonia	77.7	0.62	84.2	2057
Malaysia	72.9	0.99	82.8	2049
Maldives	78.2	1.50	94.9	2033
Moldova	72.6	0.43	76.8	2089
Mongolia	65.7	1.10	75.7	2055
Montenegro	80.7	0.39	84.9	2070
Nepal	59.0	2.53	81.7	2038
Oman	79.3	0.87	88.7	2043
Pakistan	51.6	1.53	62.8	2060
Palestine	71.8	0.70	78.6	2063
Poland	84.5	0.80	93.8	2037
Qatar	83.6	1.12	96.7	2033
Romania	79.6	0.74	87.6	2047
Russia	81.1	0.67	88.5	2047
Saudi Arabia	77.8	0.89	87.3	2045
Serbia	80.5	0.84	89.8	2042
Singapore	92.4	0.68	101.0^b^	2029
Slovakia	83.2	0.55	89.3	2049
Slovenia	90.7	0.61	98.2	2032
Sri Lanka	77.1	0.88	86.3	2046
Syria	69.9	1.02	79.8	2052
Tajikistan	60.6	0.35	63.4	2160
Thailand	76.4	0.92	86.0	2046
The Philippines	57.9	0.42	61.2	2145
Turkey	77.8	1.36	92.6	2035
Turkmenistan	68.2	0.63	74.0	2077
Ukraine	75.3	0.21	77.4	2149
Uzbekistan	67.8	0.47	72.1	2097
Vietnam	71.7	1.26	84.4	2043
Yemen	56.2	1.40	67.4	2058

Abbreviations: NCD, Non-communicable disease; SDG, Sustainable Development Goal. The global average from 2017 for 24 health-related indicators with defined targets. ^a^Based on the mean percentiles for defined targets. ^b^The score higher than 100 indicated achieving the SDG target before 2030.

 The highest score for MM was 99.8 in Estonia, and the scores were higher than 90 points in Poland (99.6), Israel (99.9), and Czech Republic and Singapore (99.5 each). The lowest score was 51.6 in Pakistan. The highest U5MR scores were 100 in Singapore, 99.8 in Slovakia, 99.5 in Czech Republic, 99.4 in Cyprus, and 99.4 in Estonia, whereas the lowest score was 68.8 in Pakistan. The highest scores for the infant mortality rate (IMR) were 100 in Cambodia, 99.3 in Indonesia, 99.2 in Laos, and 99.2 in Malaysia, whereas the lowest score was 44 in Pakistan. Association of Southeast Asian Nations (ASEAN) and Central and Eastern European countries had outstanding performance in healthy lifestyles, ranking high among the BRI countries. Pakistan had poor performance, ranking the last for these three indicators.

 For NCD mortality, six of the top 10 countries were located in West Asia. Singapore had the best performance, with a score of 100, attributed to the lowest age-standardized mortality rate for cardiovascular disease, cancer, diabetes, and chronic respiratory diseases among residents aged 30 to 70 years from 1990 to 2017. Apart from Singapore, the 10 countries with the highest scores were Israel (98.6), Kuwait (97.5), Cyprus (94.9), Maldives (94.1), Slovenia (93.8), Qatar (93.6), Bahrain (92.2), Thailand (91.5), and Jordan (91.2). Conversely, the 10 countries with the lowest scores were Afghanistan (25.1), Mongolia (48.3), Turkmenistan (54.1), Pakistan (55.0), Uzbekistan (55.8), Ukraine (56.6), Sinai Peninsula (58.3), Yemen (58.8), Azerbaijan (60.3), and Laos (62.2). China scored 83.0 and was ranked No. 23.

 The countries with the highest scores for alcohol consumption were Iran and Pakistan (99.9), Palestine (99.3), and Bangladesh (99), whereas the country with the lowest score was Ukraine (0.00). This finding indicates that alcohol abuse should be prevented in Ukraine. The government should strengthen regulations on alcohol abuse and educate the population on the negative consequences of excessive drinking. The performance in WASH mortality was high in most BRI countries, with 34 countries reaching a score of 100 and 15 countries reaching a score of 99. It is estimated that the mortality from unsafe water drinking and poor sanitation and habits will decrease significantly by 2030. With regard to health-worker density, only Slovakia and Palestine had a score >70 (74), and the other 64 countries had low scores for this indicator. Therefore, these goals should be strengthened in low- and middle-income countries.

 The highest score for healthy Lif index was 87.3 in Bhutan, and the scores were higher than 80 points in Bangladesh (83.0), and Iran (80.3). The lowest score was 28.0 in Montenegro. For the healthy P-I index, 49 out of 66 countries scored more than 90 points, with the highest and lowest score of 99.9 in Singapore and 57.3 in Pakistan. For the healthy G&D index, there are totally 27 countries and 14 countries scored over 90 points and 80 points, respectively. The highest and lowest score of G&D were 100.0 points in Cyprus and 28.5 in Yemen, respectively. The median score of the healthy Env index was 83.5 points in Syria and 84.2 points in Armenia, respectively. A total of 19 countries scored over 90 points, with the highest and lowest score in Brunei (98.5) and Afghanistan (28.7), respectively (Supplementary file 1, Figure S1).

###  Association Between Socio-demographic Index and Sustainable Development Goals 

**Figure 2:**
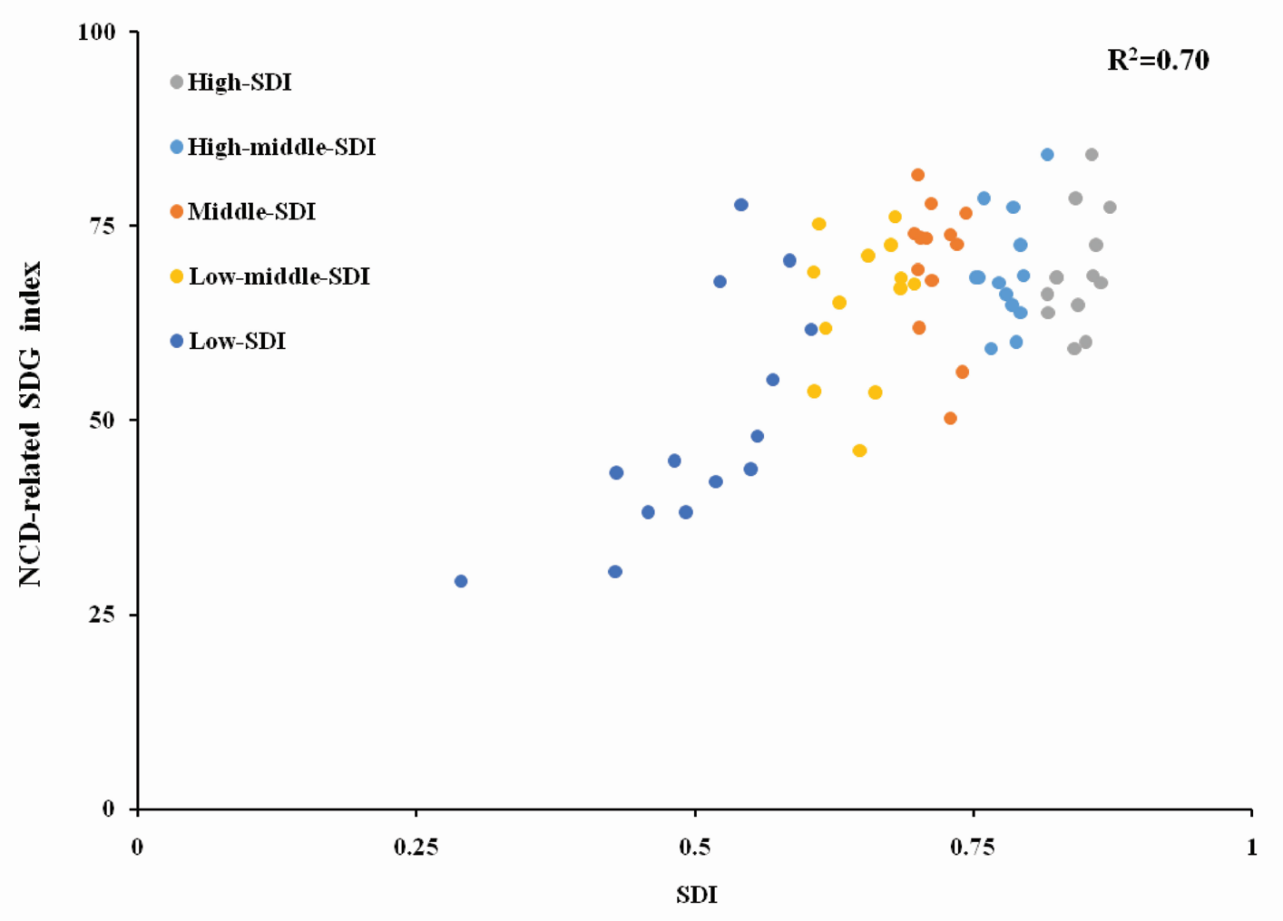
Association Between the Values of Non-communicable Disease-Related Sustainable Development Goal Indicators and Socio-demographic Index Scores. Abbreviations: NCD, Non-communicable disease; SDG, Sustainable Development Goal; SDI, Socio-demographic Index.

 The NCD-related SDG index was positively associated with SDI quintiles (R^2^ = 0.70), indicating that the higher was the SDI, the best was the health of the population (Figure 2).

 Most low-SDI countries had low SDG indexes. SDI values are shown in Table S2 (Supplementary file 1). However, among the 10 countries with the highest NCD-related SDG indexes, Palestine presented the lowest SDI, whereas the other nine countries had higher SDIs. Among the 10 countries with the lowest SDG indexes, Indonesia presented the highest SDI.

 NCD scores were positively associated with SDI quintiles. High-SDI countries had high NCD scores and low mortality (Figure 2). Overweight scores were negatively related to SDI quintiles. The scores for substance abuse were negatively associated with SDI quintiles, ie, the higher was the SDI, the higher were the rate of smoking and alcohol abuse.

###  Progress in Achieving the Targets for NCD-Related SDGs

 On the basis of past trends, most countries were projected to have higher NCD scores in 2030 than in 2017. The probability of achieving NCD targets varied substantially. The average annual rate of change from 1990 to 2017 was 1.33% (Table 2). The predicted average NCD index for 2030 was 80.4% and did not meet the SDG target (>99%). The results showed that 19 countries would have an SDG index of more than 90% by 2030, but only two countries—Cyprus and Singapore—would fully meet the target (Table 2).

 China and Slovenia are expected to reach their targets in 2031 and 2032, respectively. The average annual rate of change in Laos (2.75%), Nepal (2.53%), Cambodia (2.27%), Bangladesh (2.18%), Afghanistan (2.16%), and Bhutan (2.11%) was lower than that of other countries in the period 1990–2017. Among these countries, Afghanistan is expected to achieve the SDG target in 2053, and the other countries are expected to achieve the targets by 2041. Furthermore, several countries had difficulty in reaching the SDG 2030 targets. Ukraine (2149), Tajikistan (2160), and the Philippines (2145) would require more than 100 years to reach the target, and Georgia (2109) would require 92 years. Generally, BRI countries required additional 29 years to reach the SDG 2030 target.

 The annual rates of change required for several indicators were met or exceeded by many countries that were not in the top decile of performance from 1990 to 2017. However, only countries in the top decile of performance in this period met or surpassed the rate of change required to meet the MM rate target for 2030, and a few countries recorded the rates of change required at the global level to meet the target in 2030. U5MR and IMR were 25.8‰ and 13.7‰ in 2017, respectively, which differed from the SDG target by -0.8‰ and -1.7‰, respectively (Table 3). The annual rate of change for U5MR and IMR in the period 1990-2017 was -4.0% and -3.1%, respectively (Table 3).

**Table 3:** Forecast for 66 Countries From the Belt and Road Initiative Based on the Average Annual Rate of Change From 1990 to 2017 for Reaching Sustainable Development Goal Targets by 2030

	**SDG 2030 Target**	**Average Estimate of BRI Countries in 2017**	**Annual Rate of change Required to Reach the SDG 2030 Target**	**1990-2017 Average Annual Rate of Change**
**Goal 2: End hunger, achieve food security and adequate nutrition, and promote sustainable agriculture**				
Indicator 2.2.1: Prevalence of stunting in children under 5 years	≤0.5%	24.90%	-25.90%	-1.80%
Indicator 2.2.2a: Prevalence of wasting in children under 5 years	≤0.5%	8.20%	-19.30%	-1.30%
Indicator 2.2.2b: Prevalence of overweight in children aged 2-4 years	≤0.5%	15.70%	-23.30%	1.90%
**Goal 3: Ensure healthy lives and promote well-being for people of all ages**				
Indicator 3.1.1: Maternal deaths per 100 000 live births	<70/100 000	93.4/100 000	-2.20%	-3.60%
Indicator 3.1.2: Proportion of births attended by skilled health personnel	≥99%	89.20%	0.80%	1.90%
Indicator 3.2.1: Under-5 mortality	At least fell to 25‰	25.8‰	-0.20%	-4.00%
Indicator 3.2.2: Neonatal mortality	Decreased ³12‰	13.7‰	-1.00%	-3.10%
Indicator 3.4.1: Age-standardized mortality rates for cardiovascular disease, cancer, diabetes, and chronic respiratory diseases in people aged 30-70 years	Reduce by 1/3	442.5/100 000	-9.10%	-1.00%
Indicator 3.4.2: Self-injury age-standardized mortality	Reduce by 1/3	9.8/100 000	-8.00%	-1.80%
Indicator 3.7.1: Satisfaction rate of women of childbearing age who require modern methods of contraception for FP	≥99%	76.50%	2.00%	0.7
Indicator 3.8.1: Basic health service coverage as defined by the Health Coverage Index	≥99%	68.00%	2.90%	1.30%
**Goal 6: Ensure availability and sustainable management of water and sanitation for all people**				
Indicator 6.1.1: Risk-weighted prevalence of unsafe or unimproved water use by the population, unsafe water by SEV	≤1%	34.80%	-23.90%	-1.50%
Indicator 6.2.1a: Risk-weighted prevalence of population use without improved environmental sanitation, unsafe environmental health measured by SEV	≤1%	28.20%	-22.70%	-3.40%
6.2.1b: Risk-weighted prevalence of unhealthy habits measured by SEV	≤1%	27.80%	-22.60%	-1.50%
**Goal 7: Ensure access to affordable, reliable, sustainable, and modern energy for all people**				
7.1.2: Risk-weighted prevalence of ambient air pollution measured by SEV	≤1%	17.10%	-19.60%	-3.30%
17.19.2c: Number of death certificates in the vital registration system relative to a country’s total deaths	≥80%	37.90%	5.90%	0.60%

Abbreviations: SDG, Sustainable Development Goal; BRI, Belt and Road Initiative; SEV, summary exposure value; FP, family planning.

 Therefore, it was very likely that the targets for these two indicators would be achieved by 2030. Moreover, the annual rate of change for MM (93.4 deaths per 100 000 in 2017) necessary to reach the 2030 target was -2.2%, whereas the average annual rate of change in the period 1990-2017 was -3.6%. The annual rate of change for safe delivery (89.2% in 2017) necessary to reach the 2030 target was 0.8%, whereas the average annual rate of change in the period 1990-2017 was 1.9%.

 For water and sanitation, the annual rate of change for safe drinking water (34.8% in 2017), environmental sanitation (28.2% in 2017), and personal hygiene (27.8% in 2017) necessary to reach the 2030 target was -23.9%, -22.7%, and -22.6%, respectively, whereas the annual rate of change in the period 1990-2017 was -1.5%, -3.4%, -1.5%, respectively. For nutritional needs, the annual rate of change for stunting (24.9% in 2017), malnutrition (8.2% in 2017), and overweight (15.7% in 2017) necessary to reach the 2030 target was -25.9%, -19.3%, and -23.3%, respectively, whereas the annual rate of change in the period 1990-2017 was -1.8%, -1.3%, and 1.9%, respectively. These results show that achieving the 2030 target for the above indicators is challenging.

###  NCD-related SDGs Across Different Geographical Locations

 Mongolia presented the lowest SDG score in East Asia. The scores of four SDGs were higher than 90: malnutrition (97.7), MM (93.6), safe delivery (99.0), and WASH mortality (99.1), whereas the score for health worker density was the lowest (1.7).

 The median score considering 10 ASEAN countries of relevance to this study—Singapore, Malaysia, Indonesia, Myanmar, Thailand, Laos, Cambodia, Vietnam, Brunei, and Philippines—was 57.8. The scores of five of these countries were higher than the median, including Brunei (83.0), Malaysia (78.6), Singapore (76.4), Thailand (67.1), and the Philippines (61.9).

 The median SDG score in West Asia was 72.3 and was higher than that in East, South, and Central Asia, ASEAN countries, the Commonwealth of Independent States (CIS), and Central and Eastern Europe. The scores of seven countries—Israel, Iran, Bahrain, Palestine, Kuwait, Oman, Syria, Jordan, and Turkey—were higher than the median. The median SDG score among eight South Asian countries was 41.1, and the scores of four countries—Sri Lanka, Maldives, Bhutan, and India—were higher than the median.

 In Central Asia, the median NCD-related SDG score among five countries was 67.9. In the CIS, the scores were 73.5 (Armenia), 72.6 (Moldova), 72.6 (Russia), and 69.5 (Georgia) and higher than the median score in the CIS (69.5). The median score among the 16 countries from Central and Eastern Europe was 68.2.

 In China, the score for modern contraceptive methods was 100, which was the highest among BRI countries. In addition, 95.9% of women of childbearing age were satisfied with modern contraceptive methods in China, and the score for this indicator was close to the SDG target (100%), demonstrating China’s excellent performance in providing universal sexual and reproductive healthcare. Furthermore, the scores for WASH mortality, malnutrition, MM, safe delivery, U5MR, IMR, self-mutilation, and adolescent fertility rate were100, 95, 98, 99, 94, 94, 90, and 96, respectively. Of the 16 indicators with clearly defined goals, MM, U5MR, IMR, and safe delivery rate reached the SDG target, indicating that Chinese residents had healthy lifestyles and adequate nutritional intake. In addition, the mean scores for PM_2.5_, smoking, alcohol consumption, and health worker density were 51, 49, 66, and 29, respectively and did not meet the SDG targets to date.

## Discussion

 Although nearly all countries were projected to have higher NCD-related SDG index scores by 2030, the performance of each indicator varied by country and SDI quintile. For many indicators, the annual rate of change required to meet targets far exceeded the pace of progress achieved by any country in the recent past. However, even for SDGs with mean projected values below the 2030 target, attaining these goals by 2030 is possible by accelerating progress in the coming years. Most countries will fall short of the SDG targets if actions to prevent and treat NCDs are not implemented. These results highlight the need for the prompt and strategic implementation of programs and continued monitoring of inequalities in NCD-related SDGs within populations. In addition, countries with high NCD-related SDG indexes had higher scores for MM rate, safe delivery, U5MR, and IMR.

 Countries with high SDG indexes usually presented lower scores for childhood overweight and alcohol abuse. Obtaining high scores for the number of death certificates was a challenge in countries with the lowest SDG indexes. As populations age, all countries need to strengthen health information systems to ensure that death registration keep space with the increasing mortality of older populations. Central and Eastern Europe and the ASEAN performed better on the NCD-related SDG index than South Asia. This finding agrees with the result that the SDG index was positively associated with SDI quintiles, indicating that the higher is the education level of the population and the national GDP, the higher are NCD-related SDG index scores.

 The goals for childhood overweight were achieved in most countries except for low-SDI countries. Therefore, strengthening some interventions, including the children’s dietary education and physical exercise, is crucial. Moreover, the threshold number of 23 physicians, nurses, or midwives per 10 000 population, which was set by the WHO in 2006, is the minimum required to providing essential maternal and child health services during the MDGs era. In our study, health worker density in most countries did not meet this target. Therefore, this short-term goal can be achieved by governments by strengthening health financing in low- and middle-income countries, decreasing health expenditure, and the training and employment of health workers to ensure high-quality medical services to the world population. In addition, governments should pay attention to the regions lacking water resources, then improving the quality of drinking water. Countries with severe air pollution should make efforts to reduce this problem, advocate energy saving and emission reduction, such as reducing coal consumption and CO_2_ emissions. Countries with low scores for substance abuse should take effective measures to control unhealthy lifestyles.

 For NCDs, Afghanistan had the lowest SDG score. Although NCDs have been adequately prevented and controlled in China, which was ranked No. 23 among the 66 countries, much work still needs to be done. The number of deaths from NCDs is expected to decrease to 338 per 100 000 by 2030 in China, which also reflects the progress in preventing and controlling chronic diseases in the past few years, and indicates that national strategies such as “Healthy China 2030” and other health policies are effective. However, the number of NCD patients in China has increased rapidly in recent years, with the acceleration of industrialization, urbanization, and ageing. The GBD 2017 results showed that deaths from NCD accounted for 89.48% of the total deaths, and the disability-adjusted life year rate was 82.63% in China (vs. 62.02% elsewhere).^11^ It is well known that chronic diseases are long-term highly prevalent conditions that cause substantial economic losses, high rates of disability and death, and poverty.^34^ Currently, exposure to NCD-related risks has been increasing, wherefore future actions against NCDs are crucial.

 NCDs have a negative effect on individual health, family budget, and national employment. Furthermore, NCDs are closely associated with other SDGs. Low- and middle-income countries, and small-island countries face enormous challenges in the pursuit of sustainable development. The ongoing monitoring of the status of NCDs in BRI countries is essential to reaching SDG targets and promoting rapid public health development in these countries.

 Until 2030, most BRI countries were projected to improve their NCD-related SDG index scores, although our results indicated gaps in potential progress at the national level in this study. This indication is urgently needed to inform strategies for achieving NCD-related SDG targets, and for many countries, they have to require a faster rate of progress than has been achieved recently. Currently, most countries have formulated national action plans, which can be used to meet the targets derived from the MDGs, however, the NCD-related SDGs have not been similarly implemented in national policies.^10,11^ Therefore, it is vital that the government invests in and implements NCD-related SDG programs and continues to monitor the inequality in the population to truly fulfill the promise of not leaving no one behind, in the remaining years of achieving NCD-related SDG targets.

## Conclusion

 The health status of the population varies greatly by country. Therefore, establishing long-term cooperation mechanisms and health information platforms, and strengthening health policy communication are fundamental. In addition, improving counseling and coordination, and advice to policy-makers in bilateral health cooperation and multilateral health governance are vital. Other recommendations include (1) encouraging academic institutions and experts to share experiences on health policy research and activities; (2) strengthening the monitoring of the health condition of the population; and (3) establishing regional disease surveillance and early warning systems.

 For many indicators, the annual rate of change required to meet established targets far exceeded the progress achieved by any country in the recent past. However, the projected scores for some indicators did not reach the 2030 target but could be improved, highlighting the potential to achieve SDGs if progress is accelerated in the coming years. Countries that do not make concerted efforts to prevent and treat NCDs will fall short of SDG targets. These results highlight the need for promptly and effectively implementing programs and continually monitoring inequalities in NCD-related SDGs within populations. In addition, governments should pay attention to regions lacking water resources, and then improving the quality of drinking water. Countries with severe air pollution should make efforts to mitigate this problem, advocate energy saving and emission reduction, and reduce CO_2_ emission and coal consumption. Countries with low scores for substance abuse should take effective measures to control unhealthy lifestyles.

## Ethical issues

 This article does not contain any studies with human participants or animals performed by any of the authors.

## Competing interests

 Authors declare that they have no competing interests.

## Authors’ contributions

 SL and HL conceived and coordinated its overall structure; DD and YZ acquired data and performed statistical analyses; LC, DD and LH contributed to the writing and editing of draft; DD and LH worked closely with other authors to align the structure and develop the conclusions. AEM contributed to the overall concept of the draft. LX, SL, WF, YZ, RZ and QG contributed to the editing and revising the draft. All authors interpreted data, revised the manuscript for intellectual content, and approved the final manuscript.

## Funding

 This study was funded by the National Natural Science Foundation of China (81872721), Key Technologies Research and Development Program of China (2017YFC1310902 and 2018YFC1315305), Ningbo Health Branding Subject Fund (PPXK2018-10), Ningbo Leading Medical & Health Discipline (2022-B18).

## Supplementary files

Supplementary file 1 contains Tables S1-S2 and Figure S1.
