# Supplementary material for: Non-communicable Disease-Related Sustainable Development Goals for 66 Belt and Road Initiative Countries
Source: Int J Health Policy Manag. 2022 Oct 29;12:6172. doi: 10.34172/ijhpm.2022.6172 (PMC10125083; doi:10.34172/ijhpm.2022.6172)
Supplement: Supplementary file 1 — contains Tables S1-S2 and Figure S1. [file ijhpm-12-6172-s001.pdf]

Article title: Non-communicable Disease-Related Sustainable Development Goals for 66 Belt and Road Initiative Countries

Journal name: International Journal of Health Policy and Management (IJHPM)

Authors' information: Lin Chen[1]¶, Donghui Duan[2]¶, Liyuan Han[1,3], Lu Xu[1], Sixuan Li[2], Yuwei Zhang[4], Wei Feng[5], Qinghai Gong[2], Angela E. Micah[6], Ruijie Zhang[1,3], Shiwei Liu[7]*, Hui Li[2]*

[1]Ningbo No. 2 Hospital, Ningbo, China.

[2]Ningbo Municipal Center for Disease Control and Prevention, Ningbo, China.

[3]Department of Global Health, Ningbo Institute of Life and Health Industry, University of Chinese Academy of Sciences, Ningbo, China.

[4]Panjin Municipal Center for Disease Control and Prevention, Panjin City, China.

[5]Fenghua District Center for Disease Control and Prevention, Ningbo, China.

[6]Department of Health Metrics Sciences/Institute for Health Metrics and Evaluation, University of Washington, Seattle, WA, USA.

[7]Division of Chronic Disease and Aging Health management, Chinese Center for Disease Control and Prevention, Beijing, China.

¶Both authors contributed equally to this paper

(*Corresponding author: lihui4329@163.com & shiwei_liu@aliyun.com)

Supplementary file 1.

Table S1. List of all countries for the "Belt and Road Initiative"

Location	Countries
East Asia	China, Mongolia
ASEAN countries	Singapore, Malaysia, Indonesia, Myanmar, Thailand, Laos, Cambodia, Vietnam, Brunei, The Philippines
West Asia	Iran, Iraq, Turkey, Syria, Jordan, Lebanon, Israel, Palestine, Saudi Arabia, Yemen, Oman, The United Arab Emirates, Qatar, Kuwait, Bahrain, Greece, Cyprus, Sinai Peninsula
South Asia	India, Pakistan, Bangladesh, Afghanistan, Sri Lanka, Maldives, Nepal, Bhutan
Central Asia	Kazakhstan, Uzbekistan, Turkmenistan, Tajikistan, Kyrgyzstan
The Commonwealth of the Independent States	Russia, Ukraine, Belarus, Georgia, Azerbaijan, Armenia, Moldova
Central and Eastern Europe	Poland, Lithuania, Estonia, Latvia, Czech, Slovakia, Hungary, Slovenia, Croatia, Bosnia, Montenegro, Serbia, Romania, Bulgaria, Macedonia, Albania

Table S2. SDI values and grading status of all countries for the "Belt and Road Initiative"

SDI rank	SDI score	SDI quintiles	Country
1	0.872215248	high-SDI	Singapore
2	0.86457342	high-SDI	Cyprus
3	0.860279598	high-SDI	Slovenia
4	0.857709406	high-SDI	Estonia
5	0.856240565	high-SDI	Brunei
6	0.850980459	high-SDI	Czech Republic
7	0.84377326	high-SDI	Poland
8	0.841690487	high-SDI	Slovakia
9	0.840877452	high-SDI	Lithuania
10	0.825131484	high-SDI	Latvia
11	0.824844721	high-SDI	Croatia
12	0.816993531	high-SDI	Greece
13	0.816804322	high-SDI	Hungary
14	0.81594436	high-middle-SDI	Israel
15	0.794722025	high-middle-SDI	United Arab Emirates
16	0.791738063	high-middle-SDI	Russian Federation
17	0.79173721	high-middle-SDI	Bulgaria
18	0.788188778	high-middle-SDI	Montenegro
19	0.785593198	high-middle-SDI	Kuwait
20	0.784193905	high-middle-SDI	Romania
21	0.7790137	high-middle-SDI	Saudi Arabia
22	0.772665439	high-middle-SDI	Belarus
23	0.765715882	high-middle-SDI	Qatar
24	0.759248836	high-middle-SDI	Malaysia
25	0.75436361	high-middle-SDI	Macedonia
26	0.75179332	high-middle-SDI	Serbia
27	0.743531097	middle-SDI	Oman
28	0.740061596	middle-SDI	Ukraine
29	0.735474229	middle-SDI	Kazakhstan
30	0.729621127	middle-SDI	Lebanon
31	0.729481001	middle-SDI	Turkey
32	0.712609905	middle-SDI	Bosnia and Herzegovina
33	0.712258604	middle-SDI	Bahrain
34	0.707319288	middle-SDI	China
35	0.702021479	middle-SDI	Armenia
36	0.701169598	middle-SDI	Azerbaijan
37	0.700086759	middle-SDI	Iran
38	0.699719344	middle-SDI	Georgia
39	0.696845045	middle-SDI	Jordan
40	0.696418617	low-middle-SDI	Turkmenistan

41	0.684614242	low-middle-SDI	Albania
42	0.684276785	low-middle-SDI	Thailand
43	0.679706328	low-middle-SDI	Sri Lanka
44	0.675572758	low-middle-SDI	Moldova
45	0.661854015	low-middle-SDI	Mongolia
46	0.655286841	low-middle-SDI	Maldives
47	0.647611359	low-middle-SDI	Indonesia
48	0.629546531	low-middle-SDI	Uzbekistan
49	0.617174396	low-middle-SDI	Philippines
50	0.611084286	low-middle-SDI	Syria
51	0.606829222	low-middle-SDI	Vietnam
52	0.606646902	low-middle-SDI	Kyrgyzstan
53	0.604307711	low-SDI	Egypt
54	0.584823813	low-SDI	Iraq
55	0.569907913	low-SDI	Bhutan
56	0.555817824	low-SDI	Myanmar
57	0.550242018	low-SDI	India
58	0.541353069	low-SDI	Palestine
59	0.522612209	low-SDI	Tajikistan
60	0.518788871	low-SDI	Laos
61	0.492158484	low-SDI	Pakistan
62	0.481619391	low-SDI	Cambodia
63	0.457988721	low-SDI	Bangladesh
64	0.429504407	low-SDI	Yemen
65	0.428511471	low-SDI	Nepal
66	0.290254968	low-SDI	Afghanistan

Country	Total	Lif	P-I	Env	NCD	Suicide	Adole	UHC	WaSH	Health worker	Certified
1 Israel	84	69	99	97	99	93	95	94	100	34	83
2 Brunei	83	73	96	99	75	92	94	75	100	38	79
3 Iran	82	80	94	86	88	94	85	75	99	39	76
4 Slovakia	81	49	99	96	82	85	87	85	100	74	94
5 Malaysia	79	67	96	92	81	89	95	70	99	52	52
7 Palestine	78	71	96	79	83	98	53	68	99	74	63
6 Bahrain	78	74	97	80	92	96	93	82	99	49	60
9 Oman	77	65	96	85	85	99	95	79	99	32	58
8 Kuwait	77	61	97	85	98	100	98	87	100	34	85
12 Slovenia	76	54	100	97	94	75	99	96	100	17	92
11 Sri Lanka	76	72	96	78	88	64	90	76	98	45	73
10 Singapore	76	71	100	96	100	90	99	98	100	5	100
13 Syria	75	66	94	84	76	99	81	65	100	45	62
16 Armenia	74	47	97	84	76	88	86	74	100	34	95
15 Turkey	74	57	94	83	87	98	85	77	100	31	79
14 Jordan	74	66	95	88	91	98	86	76	100	14	73
21 RF	73	45	98	91	65	53	86	82	100	43	91
20 Moldova	73	59	94	78	69	76	87	69	100	40	88
19 Kazakhstan	73	55	95	85	65	57	83	70	100	40	86
18 Estonia	73	47	100	96	85	78	94	88	100	23	97
17 China	73	71	96	76	83	90	96	81	100	29	77
22 Latvia	72	45	98	93	72	69	91	83	100	37	97
24 Iraq	71	67	90	80	90	96	63	67	99	27	57

23 Maldives	71	66	95	80	94	98	91	78	99	21	68
27 United Arab Emirates	69	60	98	86	63	94	94	64	99	9	72
26 Kyrgyzstan	69	63	91	71	70	84	77	64	99	23	95
25 Georgia	69	44	95	80	64	89	71	67	100	38	65
37 Turkmenistan	68	60	90	80	54	87	90	63	99	10	81
36 Belarus	68	40	98	91	70	65	90	85	100	62	90
35 Tajikistan	68	74	83	63	65	93	65	52	93	53	59
34 Hungary	68	42	98	94	74	75	88	86	100	12	96
33 Bosnia and Herzegovina	68	30	98	87	79	91	95	75	100	45	78
32 Cyprus	68	54	99	97	95	95	100	100	100	5	70
31 Albania	68	35	96	86	89	94	88	81	100	36	76
30 Croatia	68	35	99	95	86	82	96	91	100	18	95
29 Macedonia	68	38	96	89	76	91	91	77	100	32	85
28 Serbia	68	35	98	89	74	79	92	81	100	45	73
40 Thailand	67	69	96	78	91	83	79	75	97	12	65
39 Greece	67	37	99	97	91	98	96	96	100	12	80
38 Poland	67	43	99	95	81	76	94	87	100	9	78
42 CzechRepublic	66	44	99	97	88	83	95	93	100	6	90
41 Saudi Arabia	66	60	97	78	87	99	96	77	99	16	53
44 Romania	65	37	97	90	73	86	79	80	100	16	89
43 Uzbekistan	65	71	92	74	56	85	81	62	99	5	84
45 Bulgaria	64	38	97	93	67	85	76	77	100	13	78
48 Egypt	62	70	91	72	58	94	62	59	97	12	52

47 Philippines	62	66	82	70	64	92	66	48	94	13	78
46 Azerbaijan	62	42	86	82	60	97	73	64	99	6	84
49 Lithuania	61	45	99	94	74	46	91	82	100	4	98
50 Montenegro	60	28	99	89	76	85	95	81	100	7	84
51 Qatar	59	60	98	78	94	95	96	85	100	4	51
52 Ukraine	56	43	96	85	57	51	85	73	100	35	89
53 Bhutan	55	87	76	73	83	93	79	57	89	43	0
55 Mongolia	54	56	90	62	48	73	85	59	99	2	71
54 Vietnam	54	64	95	70	76	90	86	68	98	46	0
56 Lebanon	50	56	94	85	82	95	64	82	99	6	0
57 Myanmar	48	77	71	53	68	94	86	49	89	50	0
58 Indonesia	46	69	82	75	68	99	84	54	81	8	0
59 Cambodia	45	66	84	58	75	96	73	54	90	19	0
60 India	44	79	75	48	69	72	85	46	62	41	1
61 Yemen	43	74	66	55	59	92	53	46	90	21	0
62 Laos	42	69	65	51	62	87	60	41	84	27	0
64 Bangladesh	38	83	68	35	73	92	54	51	85	34	0
63 Pakistan	38	80	57	50	55	96	73	39	79	7	0
65 Nepal	31	75	72	41	73	88	61	50	71	7	0
66 Afghanistan	29	79	62	29	25	86	40	31	92	4	0

Value 100 75 50 25 0

Figure S1. Scores of 12 non-communicable disease-related sustainable development goal indicators in 66 countries from Belt and Road Initiative countries in 2017.
